# ASO Author Reflections: A New Era for Minimally Invasive Surgery in Liver Cancer

**DOI:** 10.1245/s10434-026-19115-0

**Published:** 2026-01-26

**Authors:** Christine E. Haugen, Nathaly Llore, Jason S. Hawksworth

**Affiliations:** 1 Department of Surgery, University of Cincinnati, Cincinnati, OH USA; 2 Department of Surgery, Columbia University Irving Medical Center, New York, NY USA

## Past

The rising incidence of primary liver cancer and mortality rates has led to continued refinements in operative approaches. While the majority of hepatectomies are open, the focus has shifted to minimally invasive techniques that provide superior clinical outcomes without compromising oncologic principles. Robotic surgery, with improved dexterity and visualization, has a role in treating liver cancer with promising results.^1^ International consensus guidelines for laparoscopy and robotic surgery in liver oncology have been described for safe practices.^2^

## Present

For centers to transition to robotic hepatectomy for primary liver cancer, an understanding of meaningful data, patient selection, and technical feasibility is necessary. Large single-center studies show that robotic liver surgery is associated with decreased blood loss and shorter length of stay without a significant difference in overall survival or oncologic margins compared with open surgery for hepatocellular carcinoma (HCC) and intrahepatic cholangiocarcinoma.^3^ Similarly, recent studies have shown that laparoscopic and robotic hepatectomy for resectable HCC have similar postoperative outcomes and overall survival. Difficulty scoring systems (DSS) and their application to laparoscopy and robotics allow an accurate prediction of case difficulty and the context in which it should be performed. Early consensus guidelines support the advantages of robotic technology in minor and major hepatectomies for liver malignancy.^2^ The Tampa DSS addresses the need for a robotic specific DSS but remains to be validated.^4^ Robotic hepatectomy offers a safe approach to higher-risk patients that are older and/or have cirrhosis or large tumors, albeit with similar rate of complications inherent to a higher-risk population.

## Future

A review of operative approaches to liver cancer supports a safe transition to robotic surgery.^5^ The wide adoption of robotic hepatectomies necessitates defining robotic DSS, multicenter studies for oncologic outcomes, and training systems to overcome the technical difficulties of minimally invasive liver resections. As the platform improves with tactile feedback and enhanced precision, its use will be expanded for highly complex tumors and cases with vascular or biliary reconstructions.
